# Larval transcriptomic response to host plants in two related phytophagous lepidopteran species: implications for host specialization and species divergence

**DOI:** 10.1186/s12864-018-4589-x

**Published:** 2018-04-18

**Authors:** M. Orsucci, P. Audiot, F. Dorkeld, A. Pommier, M. Vabre, B. Gschloessl, S. Rialle, D. Severac, D. Bourguet, R. Streiff

**Affiliations:** 1 CBGP UMR 1062, INRA-IRD-CIRAD-Montpellier SupAgro, Montferrier sur Lez, Montpellier, France; 2 DGIMI UMR 1333, INRA-Université de Montpellier, Montpellier, France; 3 MELGUEIL DIASCOPE UE 0398, INRA, Mauguio, France; 4 MGX-Montpellier GenomiX, c/o Institut de Génomique Fonctionnelle, Montpellier, France; 5 Present address: Department of Ecology and Genetics, EBC, Uppsala University, Norbyvägen 18D, 75236 Uppsala, Sweden

**Keywords:** Specialization, Plant-insect interactions, Species divergence, Plasticity, Gene expression, Microorganisms

## Abstract

**Background:**

Most phytophagous insects have morphological, behavioral and physiological adaptations allowing them to specialize on one or a few plant species. Identifying the mechanisms involved in host plant specialization is crucial to understand the role of divergent selection between different environments in species diversification, and to identify sustainable targets for the management of insect pest species. In the present study, we measured larval phenotypic and transcriptomic responses to host plants in two related phytophagous lepidopteran species: the European corn borer (ECB), a worldwide pest of maize, and the adzuki bean borer (ABB), which feeds of various dicotyledons. Our aim was to identify the genes and functions underlying host specialization and/or divergence between ECB and ABB.

**Results:**

At the phenotypic level, we observed contrasted patterns of survival, weight gain and developmental time between ECB and ABB, and within ECB and ABB reared on two different host plants. At the transcriptomic level, around 8% of the genes were differentially expressed (DE) between species and/or host plant. 70% of these DE genes displayed a divergent pattern of expression between ECB and ABB, regardless of the host, while the remaining 30% were involved in the plastic response between hosts. We further categorized plastic DE genes according to their parallel or opposite pattern between ECB and ABB to specifically identify candidate genes involved in the species divergence by host specialization. These candidates highlighted a comprehensive response, involving functions related to plant recognition, digestion, detoxification, immunity and development. Last, we detected viral, bacterial, and yeast genes whose incidence contrasted ECB and ABB samples, and maize and mugwort conditions. We suggest that these microorganism communities might influence the survival, metabolism and defense patterns observed in ECB and ABB larvae.

**Conclusions:**

The comprehensive approach developed in the present study allowed to identify phenotypic specialization patterns and underlying candidate molecular mechanisms, and highlighted the putative role of microorganisms in the insect-host plant interaction. These findings offer the opportunity to pinpoint specific and sustainable molecular or physiological targets for the regulation of ECB pest populations.

**Electronic supplementary material:**

The online version of this article (10.1186/s12864-018-4589-x) contains supplementary material, which is available to authorized users.

## Background

The high degree of species diversity observed in phytophagous insects probably results from their propensity to specialize on different host plants. Host specialization can lead to the emergence of new species, when, for example, host fidelity promotes spatial or temporal isolation of insect populations [1]. Two major and non-exclusive processes are thought to underlie the so-called ‘speciation-by-specialization’ in phytophagous insects: phenotypic plasticity and host specialization by local adaptation. Phenotypic plasticity, i.e. the capacity of a single genotype to produce different phenotypes in response to varying environmental conditions, facilitates speciation by generating phenotypic variation within species [2]. Divergent selection, associated to different resources, then reinforces phenotypic and genetic differences between specialized populations. Any mechanism coupling adaptive divergence to reproductive isolation will promote the formation of new species adapted to specific host ranges [3–6].

Host plants are key components of the ecological niche of herbivorous insects, through their roles as a food resource, overwintering refuge, oviposition site and, in some cases, mating site. By consequence, the use of plant hosts involves key adaptations in behavioral traits (e.g. host plant choice [7, 8]), morphological traits (e.g. variations in mouthparts according to the pollination mechanism [9]) and physiological traits essential for development and survival (e.g. enzymatic activities for the detoxification of some secondary plant compounds [10, 11]). At the molecular level, Simon et al. [12] reviewed mechanisms involved in specialization to the host plant in lepidopterans and hemipterans, and highlighted the importance of genes relating to sensing, digestion and detoxification. In lepidopterans, plasticity and host specialization are related to three principal groups of gene functions: (1) the expanded family of genes encoding olfactory and gustatory receptors involved in host sensing [13]; (2) variation of the specificity and expression of gut proteinases involved in digestion of primary plant compounds [14]; and (3) the activity of glucosinolate sulfatases, P450 enzymes and carboxylesterases involved in the detoxification of some plant compounds or xenobiotics [10, 15–17]. In addition to sensing, digesting and detoxifying genes, whole-transcriptome surveys of phytophagous moth and butterfly larvae reared on various host and non-host plants revealed differences in the expression of structural and developmental genes [18, 19] or in ribosomal activity [20]. RNAseq analyses of this type, without prior assumptions, should therefore add to our knowledge of the complex plastic responses of lepidopteran larvae to the host plants on which they feed.

Until recently, *Ostrinia nubilalis* Hübner (Lepidoptera: Crambidae), the European corn borer (ECB), was considered to be polyphagous [21], with a host range including a crop recently introduced into Europe, the maize (*Zea mays* L.), on which it has become a major pest worldwide. In the last decades, two genetically differentiated host races of ECB have been identified in Western Europe, one feeding mostly on maize (*Zea mays* L.), and the other feeding on various dicotyledons, including mugwort (*Artemisia vulgaris* L.) and hop (*Humulus lupulus* L) [22–24]. After reviewing phenotypic, behavioral and genetic data, Frolov et al. [25] suggested that these two taxa should be separated into sibling species, with populations feeding on maize classified as *O. nubilalis* (ECB), and those feeding on dicotyledons belonging to *O. scapulalis* (ABB for adzuki bean borer). Several independent studies have characterized specialization of ECB on maize and ABB on mugwort for two key components of the life cycle: larval performance and oviposition choice. These studies highlighted that the specialized traits differ in nature and strength between ECB and ABB: while ECB shows a strong preference for maize (coupled with an avoidance of mugwort) during oviposition [7, 26], its larval performance is slightly better [27] or equivalent [26] on maize than on mugwort. In contrast, ABB larval survival is significantly lowered on maize compared to on its usual host, the mugwort. During oviposition, ABB females slightly prefer mugwort to maize, but do not reject maize plants in no-choice conditions. Let note that ECB and ABB larvae all gain more weight when developing on maize than on mugwort and weight is usually considered as positively correlated to fitness, e.g., for example by a higher reproductive success of adult moth issued from heavier larvae [28]. This trait may thus compensate the lower larval survival observed in ABB larvae, but this has not been evaluated in this species.

An evolutionary scenario has been proposed in which ECB and ABB diverged independently from a common ancestor living on wild dicotyledons (and probably on mugwort, which is native to Eurasia [29]). During or after the species divergence, ECB shifted on, and specialized to, maize after its introduction and the beginning of its cultivation in Europe ca. five hundred years ago [30]. All of the above behavioral and physiological patterns suggest that ECB colonized and specialized to the maize through an aptitude to develop on this crop at the larval stage, and a high capacity to recognize this suitable host at the adult stage. By contrast, the weak ABB survival on maize suggests that this species did not acquire the optimal capacities to use and detoxify this exotic plant.

**Fig. 1:**
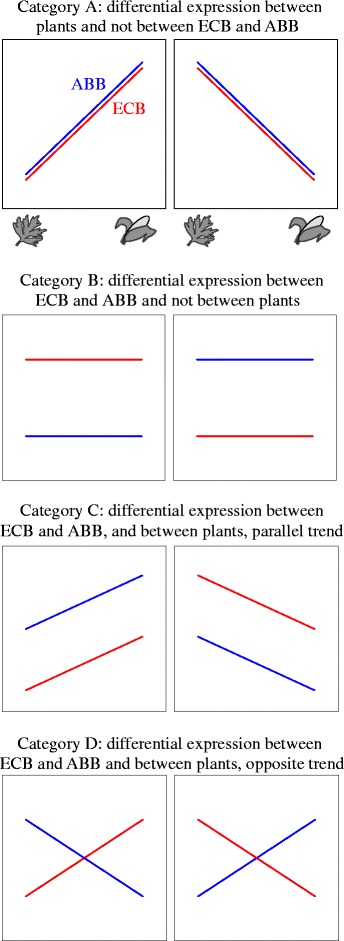
**a-d**: Schematic classification of differentially expressed (DE) genes into four evolutionary categories based on expression patterns between species and between environments (plants). Mean gene expression for ABB (blue lines) and ECB (red lines) is illustrated on the *y*-axis for samples reared on maize or mugwort (plant host indicated on the *x*-axis)

In the present study, we aimed at testing this scenario by investigating the mechanisms underlying the contrasting patterns of specialization of ECB and ABB at the larval stage. For that, we measured differential gene expression in ECB and ABB larvae reared on native and alternative host plants and identified candidate genes involved in host specialization and/or in ECB-ABB divergence. We classified differentially expressed (DE) genes in various categories according to their plastic or divergent pattern (Fig. 1). These categories allow to distinguish the genes that diverged between ECB and ABB independently of host specialization, from those which diverged because of the host shift towards maize.

In addition, we identified in DE genes sequences homologous to viruses, yeasts and bacteria. While these microorganisms were not targeted in the initial design, their presence and diversity revealed interesting patterns of differentiation between ECB and ABB samples, and between native and alternative host plants, that highlighted their potential role in the divergence of ABB and ECB and in the host specialization.

The comprehensive approach proposed here, combining a focus on specialization *patterns* and the discovery of candidate molecular *mechanisms*, is an important step for pest management. The evolutionary context of the ECB-ABB species complex is indeed a textbook example to test a sustainable control of pest populations with a limited impact on non-target species [31]. ECB and ABB are sympatric and share a recent common ancestry, while the first became a pest specialized onto maize, and the second remained plastic and polyphagous. These evolutionary and agronomic contexts offer the opportunity to identify the best and more sustainable molecular or physiological targets for the regulation of ECB populations while limiting the effects on non-target fauna.

## Methods

### Experimental design and life history traits analysis

**Fig. 2:**
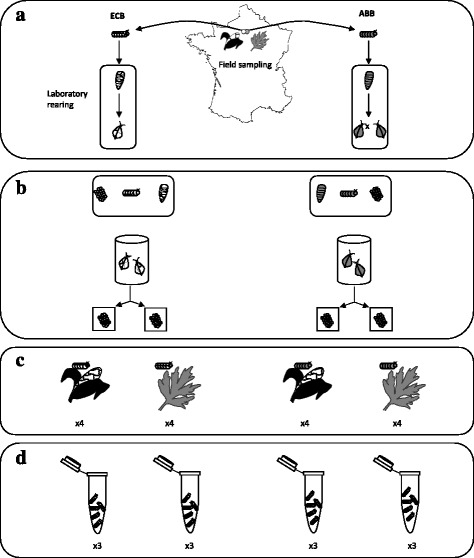
Experimental design of the reciprocal transplant experiment and sequencing. **a**. Diapausing larvae were collected from maize stands for ECB and from mugwort stands for ABB and reared in the laboratory until the adult stage. France map was downloaded from the ‘map’ package from the R software. **b**. Male and female adults (about 10 in total) were placed in breeding cages and allowed to mate freely. Their progeny was reared to pupation on an artificial diet. After emergence, single mating pairs were established. The progeny of mating pairs that laid more than 10 egg masses, was split into equivalent batches before plant infestations in the various experimental set-ups and replicates. **c**. Mature egg masses were placed on the bottom side of leaves, in insect-proof cages (four replicates of each “moth species x plant” set-up) in outdoor conditions in the South of France, until the fourth instar (L4) was obtained. The larvae were then sampled, weighed, flash-frozen in liquid nitrogen, and stored at − 80 °C until RNA extraction. **d**. For three replicates of each set-up, total RNA was extracted and equimolar pools of RNA from about 20 larvae were sequenced on a HiSeq2000 (Illumina, 2 × 100 bp)

In this study, we used larval samples from a previous reciprocal transplant experiment in which we characterized several life history traits, including survival and weight (see [26] for details). This experiment involved controlled infestations of maize and mugwort plants with fertile egg masses from the two moth species: ECB and ABB (Fig. 2). These egg masses were obtained in our laboratory by rearing moths collected in the field, near Versailles (48°48′19″N, 2°08′06″E, France) in 2013. Controlled infestations were established in insect-proof outdoor cages filled with maize or mugwort plants. We deposited 24 egg masses per cage of maize and 20 egg masses per cage of mugwort. The complete experimental design was as follows: 2 moth species × 2 host plants and four replicates per experimental set-up (i.e. moth species x plant). Larval development took place over two to three weeks and survival was estimated as the ratio of the number of larvae collected at the sampling date over the initial number of eggs deposited. All collected larvae were weighted before storage at − 80 °C.

In the present study, we reanalyzed weight gain specifically for the larvae used for the subsequent sequencing, that represent a subset of the samples studied in [26]. In addition, we present new data on their developmental time from egg to the fourth larval instar. Statistical analysis was performed with the R software [32]. We used different generalized linear models (GLM), depending on the distribution of the residuals for each quantitative trait. The complete model contained the following explanatory variables: (1) the species of plant on which the individual developed, (2) the moth species, (3) the sex of the insect, and their interactions. The third variable was used only for the analysis of the weight variable because previous studies on ECB showed that females were heavier than males [27]. We then performed a model selection by removing the non-significant terms (*P* > 0.05) beginning with highest-order terms using likelihood ratio tests (LRT [33, 34]) and a contrast analysis on factor levels for explanatory variables. The non-significant levels (*P* > 0.05) were grouped together (LRT, [34]).

### RNA sequencing

Fourth-instar larvae (L4) were sampled, weighed and stored at − 80 °C. We extracted total RNA from each L4 with the AllPrep DNA/RNA 96 Kit (Qiagen), according to the manufacturer’s protocol. RNA was quantified with a Nanodrop spectrometer, and equimolar amounts of individual extracts were pooled to obtain 12 samples corresponding to the three replicates of the four experimental set-ups (Fig. 2). All but one of the pools contained extracts from 20 L4 (19 for replicate 1 of ECB on maize). The sex of each larva had previously been determined by multiplex PCR amplification [26]. The observed sex ratio was 1:1 for all but one of the pools (Additional file 1: Table S1).

The 12 pooled RNA extracts were sent to the MGX platform (Montpellier, France) for sequencing. Tagged cDNA libraries were constructed with the TruSeq Stranded mRNA Sample Preparation Kit (Illumina) according to the manufacturer’s protocol, and paired-end 100 bp sequencing was performed on three lanes of an Illumina HiSeq 2000. Each lane contained four libraries. To avoid confounding effects between lane and experimental set-up, the three replicates of a same experimental condition were separated and sequenced into different lanes (one replicate per lane).

### Data processing

After sequencing, all reads were subjected to quality control and trimming with Trimmomatic v0.25, to remove Illumina sequencing adapters and low-quality reads [35]. After quality control, the ribosomal and bacterial contaminants were removed with SortMeRNA v2.0 [36], which takes as input the files containing all high-quality reads and multiple rRNA database files (16S and 23S for bacteria and archae and 18S and 28S for eukarya) to sort apart in two files rRNA and rejected reads (corresponding to mRNA).

### De novo transcriptome assembly and automatic annotation

We used the Trinity program suite (with default parameters and read normalization) to assemble the six ABB libraries and the six ECB libraries into two separate assemblies [37]. We then combined HiSeq data with 454 data previously obtained for ECB and ABB (details available from [38]). We used the EMBOSS splitter program to create overlapping subsequences (length 300 bp, overlap 200 bp) for each of the 454 transcripts of the HiSeq transcript sets. All 454 and HiSeq subsequences were then assembled with Newbler ([39], 454 Life Sciences Corporation, v2.9), with a minimum overlap of 40 bp, a minimum overlap identity of 97% and the ‘Het’ (for heterozygote) options. Newbler has the option, compared to other assemblers, to reconstitute alternative transcripts of a same unigene (called isotigs and isogroup respectively in Newbler outputs). We selected the longest transcript (isotig) in each isogroup as representative of each unigene to eliminate possible bias in the expression analyses due to alternative transcripts.

As the statistics for 454-HiSeq ‘hybrid’ assemblies were better than those for Trinity assemblies (in terms of the number and length of transcripts) we retained these ‘hybrid’ transcriptomic references, one for ABB and another for ECB, for subsequent analyses. The two final sets of genes (one for ABB and the other for ECB) used as references are referred to hereafter as ‘ABB-ref’ and ‘ECB-ref’. We finally evaluated the quality of ABB-ref and ECB-ref using BUSCO (v3.0.2 [40]) analysis that searched in reference transcript sets (parameter -t tran) for the presence of 1066 conserved arthropod coding-genes (downloaded from http://busco.ezlab.org/v2/datasets/arthropoda_odb9.tar.gz in January 2018).

For functional and GO-term annotations, ECB-ref and ABB-ref genes were compared to NCBI nr (version 2015–04-25) and UniprotKb ([41] version june 2015), with *blastx* BLAST [42] and InterProScan [43].

BLAST analysis revealed that some of the genes probably originated from organisms other than lepidopterans or insects (e.g. microorganisms, see results). We then rechecked the raw reads specifically for the presence of bacterial sequences because these latter had been filtered out before assembly of the ABB-ref and ECB-ref sequences. For that, raw reads were mapped on a subset of bacterial sequences extracted from the NCBI database and counted with the procedure detailed below for ECB-ref and ABB-ref.

### Gene expression in ECB and ABB samples

We mapped raw reads onto ABB-ref and ECB-ref with Bowtie2 ([44]; v2.2.4), using the default parameters and the “sensitive” option.

The number of mapped reads per gene was determined with SAMtools ([45]; version 1.2). Gene-specific variations (dispersion and logarithmic fold-changes in reads counts between experimental conditions) were estimated with the Bioconductor ‘Edge R’ package ([46]; version 3.8.6). A negative binomial distribution was fitted to count variations, and genes with less than 40 mapped reads in all conditions were excluded from the analysis. Reads counts were normalized for library size and for the total number of genes expressed in each sample, with the EdgeR function *calcNormFactors*, using the trimmed mean of M values (TMM) method [46].

Variations in gene read counts (used as a proxy for the level of gene expression) were analyzed with generalized linear models (GLM), as implemented in EdgeR, to estimate plant and moth species effects. For each gene read count, *g*_*i*_, a likelihood ratio test (LRT) was used to compare a full model (*g*_*i*_ = *plant species + moth species + plant species* x *moth species + ε*) and a reduced model from which the interaction was removed (*g*_*i*_ = *plant species + moth species + ε). P*-values were adjusted for multiple testing by the Benjamini-Hochberg procedure [47], with a false discovery rate (FDR < 0.01).

We used these different statistical effects to classify genes into four categories according to their relative reaction norms between ECB and ABB, and between maize and mugwort (Fig. 1): (A) genes displaying differential expression between environments (plants) but not between species; these genes are involved in the phenotypic plasticity common to ECB and ABB, (B) genes displaying differential expression between moth species but not between plants; these genes are involved in species divergence unrelated to the larval host plant specialization, (C) genes displaying differential expression both between plants and between moth species, following parallel trends; these genes are involved in adaptive phenotypic plasticity, i.e. in plasticity that evolved between ECB and ABB and (D) genes displaying differential expression between environments (plants) and between moth species, but with opposite trends; these genes are involved in the species divergence related to host plant specialization. Let note that category C may also reflect similar stress responses in ECB and ABB to one of the two plants, and category D stress response induced by being reared on non-host plants.

Blast2GO (v2.5, database Sep2015, [48]) was used to implement Fisher’s exact tests to assess the enrichment of these four categories of genes in particular GO terms. We identified under- and over-represented GO categories after a correction for multiple testing (FDR < 0.05).

## Results

### Life history traits and host plant specialization in ECB and ABB.

**Fig. 3:**
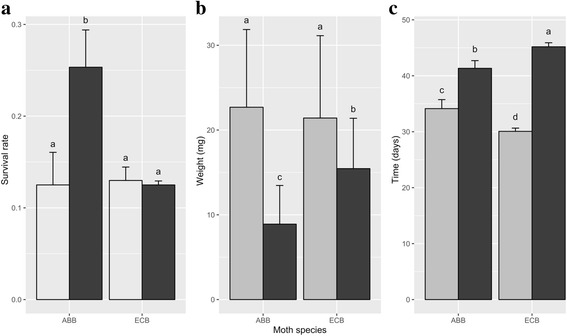
Life history traits of ABB and ECB reared on maize (light gray) or mugwort (dark gray). Mean larval survival (**a**), mean L4 weight (**b**) and mean development time from egg to L4 (**c**) presented with their standard errors. Different letters above the bars indicate significant differences (*P* < 0.05). Note: the data used in (**a**) have been published (Orsucci et al. 2016), whereas the data in (**b**) and (**c**) have been reanalyzed here for the L4 samples used in the sequencing pools (*N* = 20)

The specialization pattern showed contrasted results between ECB and ABB larvae. First, larval survival rates were similar for ECB on the usual (maize) and alternative (mugwort) hosts, whereas, for ABB larvae, survival rates were higher on mugwort (usual host) than on maize (alternative host; Fig. 3a [26]). ECB and ABB larvae feeding on maize were significantly heavier than those feeding on mugwort (Chi^2^ = 28.35, df = 1, *P* < 0.001; Fig. 3b). In addition, we found a significant effect of sex (Chi^2^ = 9.65, df = 2, *P* < 0.01), and a significant interaction between moth species and plant species (Chi^2^ = 29.27, df = 1, *P* < 0.001). Developmental time (from eggs to L4) was significantly shorter on maize than on mugwort, for both ECB (F = 4981.2, df = 1, *P* < 0.001) and ABB larvae (F = 1565.5, df = 1, *P* < 0.001, Fig. 3c). Yet, the ABB larvae developed slower on maize than did ECB larvae (F = 489.53, df = 1, *P* < 0.001).

### *De novo* assembly and annotation

Paired-end Illumina sequencing of six ABB libraries yielded 225,485,243 raw reads. After quality filtering 195,233,078 of these reads were assembled into 100,016 transcripts corresponding to 60,844 unigenes with a mean sequence length of 810 bp. For the six ECB librairies, 220,397,770 raw reads were obtained after sequencing and after quality filtering, 164,414,432 ECB reads were assembled into 96,314 ECB transcripts corresponding to 59,720 ECB unigenes with a mean sequence length of 919 base pairs (bp). The ‘hybrid’ ABB and ECB assemblies, including 454 and Hiseq transcripts, yielded 31,355 ABB and 30,108 ECB transcripts with a mean length of 1656 bp and 1636 bp, respectively, covering about 52 Mb in ABB and 49 Mb in ECB. Taking the longest sequence as the sole representative for each unigene yielded 19,320 ABB-ref and 18,966 ECB-ref genes. Within the ABB-ref transcript set, 91.2% of the arthropod conserved BUSCO gene set was identified, 83.1% of which were complete. The set of ECB-ref transcripts held 89.5% of the conserved arthropod genes, 82.6% of which were assembled at full-length.

Search for homology in nucleotide and protein sequence databases yielded significant hits for 67% (ABB-ref) and 67.7% (ECB-ref) of the genes, 54.5% (ABB-ref) and 54.9% (ECB-ref) of which had an associated GO term. Most of the annotated genes were homologous to lepidopteran genes or to other insect orders. However, we observed ‘alien’ genes from plants and microorganisms potentially associated to Lepidoptera. The observation of genes attributed to plant homologs was not surprising, given that the L4 were frozen immediately after sampling, without starvation or gut cleaning. Beside plant residues, we also detected viral, yeast and bacterial sequences. While the target of the sequencing was the expression of moth larvae genes, we did not filtered out these alien genes from the following analyses because of their potential importance in the defense response and more generally in the metabolism of ECB and ABB larvae (more details in the results section “Variation in expression of microorganisms’ genes”).

### Reads mapping in both references for ECB and ABB samples reared on maize or mugwort

The percentage of reads mapping in the ABB-ref and ECB-ref sequences ranged from 64.64% to 69.06% (Additional file 1: Table S2). Filtering out genes with coverage inferior to 1× (i.e. 100 bp x read number for one gene / length of the gene), yielded a total of 19,320 expressed transcripts for the ABB-ref and 18,966 for the ECB-ref. Most of these expressed transcripts (18,016 for ABB-ref and 17,076 for ECB-ref, Additional file 1: Figure S1) were shared between all experimental set-ups, Additional file 1: Figure S1).

**Fig. 4:**
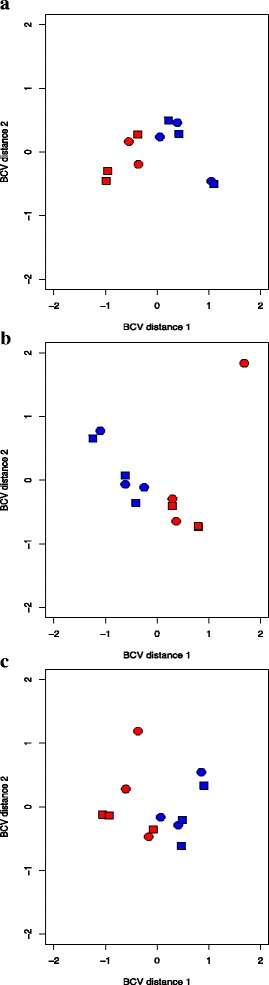
Multidimensional scaling plot of reads counts per library. Barycenters are plotted in red for the ECB and in blue for ABB. The samples on maize are represented by squares and those on mugwort by circles. **a** and **b** plots present all replicates after filtering and normalization in ABB-ref and ECB-ref, respectively. **c** present replicates in ECB-ref without one outlier replicate (ECB-mug2)

Multidimensional scaling (MDS, Fig. 4) plots of the normalized counts in ECB-ref and ABB-ref clearly separated the samples by ECB vs. ABB species. No clear discrimination between experimental conditions (i.e. host plants) appeared on the first two main axes, suggesting that *i)* most of the expressed genes do not respond to these conditions, *ii)* the experimental effect is largely weaker than the species effect. Besides, the multidimensional plot in ECB-ref showed that one ECB sample reared on mugwort (ECB_Mug2, Fig. 4) was a strong outlier, for unknown reason. We thus chose for clarity to illustrate the following analyses with the ABB-ref. The results for ECB-ref are nevertheless presented in Supplementary Materials, as well as a comparison between the analysis with all replicates in ECB-ref, and with all but this outlier ECB sample (Additional file 2: Table S3).

### Differentially expressed (DE) genes in ECB and ABB samples reared on maize or mugwort

#### Overall expression and ‘plant’, ‘species’ and ‘interactions’ effects

In total, 1525 of 19,320 ABB-ref genes (8%) displayed significant differential expression between experimental conditions and/or between ECB and ABB, according to the GLM full model. Most of this differential expression was due to the moth species effect, with 1213 ABB-ref genes displaying a significant effect of species in the reduced model. An interaction effect was detected for 254 ABB-ref genes, and a plant effect was detected for 327 ABB-ref genes. Further information about the DE genes, including count variations in ECB and ABB on mugwort and maize, are provided in Additional file 3: Table S4, together with their best BLAST hit and GO annotations.

#### Categorization of DE genes and functional patterns

**Fig. 5:**
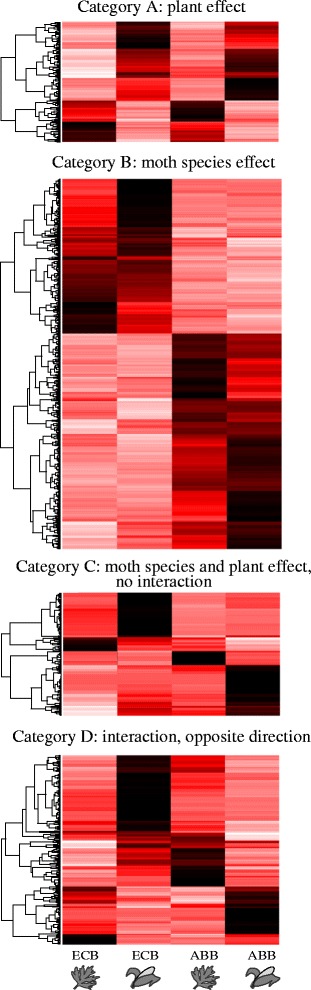
**a**–**d** Heatmaps of DE ABB-ref genes classified into the four evolutionary categories as defined in Fig. 1. Genes (rows) were clustered with the ‘hclust’ algorithm of the ‘heatmap’ function (R software). A white-red-black color scale indicates the low-middle-high expression of the genes

We classified the ABB-ref DE genes into four categories according to their relative reaction norms (Fig. 1a-d) in ECB and ABB samples reared on maize or mugwort (Fig. 5a-d, and supporting information Additional file 1: Figure S2 for ECB-ref). We found that most of the DE genes were in the B category (985 DE genes over a total of 1503) that represented the class of genes with divergent pattern of expression between ECB and ABB whatever the host plant used during larval development. In this category, we identified 32 over- and 25 under-represented GO terms at a 0.05 FDR threshold, mostly related to DNA and organic substances metabolism (Additional file 1: Table S5).

DE genes with expression varying between experimental conditions (host plants) were evenly distributed in the A, C, and D categories (161, 125 and 232 DE genes respectively). Because these categories contained few DE genes, and even fewer associated GO terms, Fisher’s exact test failed to identify enriched GO categories after FDR correction for multiple testing, except for one GO term overrepresented in the C category (nutrient reservoir activity). Qualitative inspection of annotated DE genes allowed, however, to identify some relevant patterns in each evolutionary category.

The genes expressed differentially between maize and mugwort but similarly in ABB and ECB samples (category A; Fig. 5a, Additional file 3: Table S4) were potentially involved in lipid, glucid and protein digestion and transport (serine proteases, lipases and glucose dehydrogenase, transporters etc.), and were, for the most, more expressed on maize than on mugwort. Immune genes were also observed in this category (cecropin, attacin, gloverin) and more expressed on maize than on mugwort. A small number of chemosensory and cuticular proteins were also identified here, and were either more or less expressed on maize than on mugwort. Finally, various genes potentially involved in development or cellular processes were detected.

Genes differentially expressed between ECB and ABB and between mugwort and maize, with similar trends in the two species (category C; Fig. 5c), were potentially involved in digestion and storage of protein, glucids and lipids, in detoxification (P450) and in cuticular processes (Additional file 3: Table S4). A majority of these genes were more expressed on maize than on mugwort in ECB and ABB samples. This category was also characterized by the presence of yeast and virus genes, mostly observed in ABB samples reared on maize for the former, and more observed in ECB samples on maize for the latter.

Finally, genes displaying significant differential expression between mugwort and maize and opposite patterns in ECB and ABB (category D, Fig. 5d) were mostly involved in cuticular processes (more expressed in ECB larvae reared on maize and less expressed in ABB larvae reared on maize), and to a lesser extent in digestion, detoxification, immunity and chemosensory processes. This category also contained genes from yeasts, most of which were more expressed on maize in ABB and less expressed on maize in ECB.

#### Variation in expression of microorganisms’ genes

**Fig. 6:**
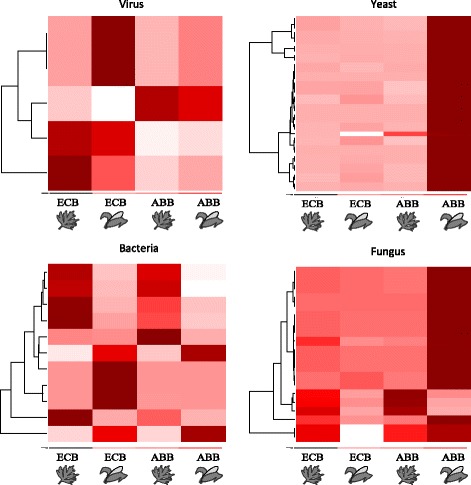
Heatmaps of DE genes homologous to virus, yeast, bacteria and fungus sequences. Genes (rows) were clustered with the ‘hclust’ algorithm of the ‘heatmap’ function (R software). A white-red color scale indicates the low to high expression of the genes

While they represent a low percentage of the data, some genes homologous to viral, fungal, yeast and bacteria were significantly differentially expressed between ECB, ABB or conditions in the GLM procedure. Detailed information about these DE genes, including their relative expression in ECB and ABB on mugwort and maize, and BLAST annotations are provided in Additional file 4: Table S6 and Additional file 5: Table S7. Because we did not target these organisms and their effects during the experiment, we have no evidence about their origin (environment or specific association to ECB and ABB), and no estimation of their putative pathogenicity or beneficial effects. However, we observed noticeable differences between ECB and ABB species, and between samples on maize or on mugwort (Fig. 6): *i)* virus and bacteria genes were more expressed in ECB than in ABB samples while yeast and fungi genes were more expressed in ABB samples than in ECB samples, *ii)* the expression of yeast and fungi genes in ABB samples reared on maize was largely higher than on mugwort.

## Discussion

Studies on phytophagous insects have regularly reported the importance of the host plants and diet on various life history traits [49–53]. In the present study, we confirmed *i)* the specialization of ECB and ABB to their respective hosts as already observed in former studies [26, 27] and *ii)* their contrasted physiological response in terms of survival, weight gain and developmental time. Maize appeared as a richer diet than mugwort for ECB and ABB larvae, as illustrated by the higher growth and faster development on this plant. These contrasted traits and tradeoffs helped identifying functional differences between ECB and ABB when reared on usual and non-usual hosts.

For the first time in this species pair, we identified the sets of genes with differential expression between ECB and ABB larvae reared on their native plant or an alternative one and classified these DE genes in plastic and/or divergent categories. We found that around 8% genes displayed significant differential expression between ECB and ABB samples and/or between samples reared on mugwort and on maize. This percentage is of the same order of magnitude as those reported in related studies (2.7% of DE genes between highly differentiated allopatric populations of *Drosophila mettleri* reared on different host plants and an artificial diet; [54], 3.9 to 11.9% of DE genes in pea aphid host races reared on their respective native hosts and on an alternative host; [55]).

### DE genes, plasticity and species divergence

The evolutionary categories A to D (Fig. 1), defined by the relative reaction norms in ECB and ABB larvae reared on maize or mugwort, summarize the divergence between ECB and ABB independent (Fig. 1b) or dependent (‘plastic genes’ Fig. 1a, c, d) of their response to the host. As previously reported [54, 55], we found few plastic compared to evolved genes in the two species, with 70% of the ABB-ref DE genes belonging to category B, and 30% of genes considered as plastic (categories A + C + D; Fig. 5). Assuming a host shift in ECB from an ancestral host plant (‘mugwort like’, i.e. a European dicotyledon species) to maize (a recently introduced plant), genes displaying significant and opposite interactions between host plant and moth species (Fig. 1d) may be the most relevant to speciation as they relate to species-specific responses to the host plant. Looking at correlations with the phenotypic trends presented above, we can hypothesize that category D contains genes related to differential survival on mugwort and maize, because opposite survival trends were observed between ECB and ABB (Fig. 3a). By contrast, categories A and C should contain plastic genes related to weight and development, as these two traits followed parallel trends in ECB and ABB (Fig. 3b-c). Functional categories related to sensing, metabolism (digestion and detoxification), development and immunity were distributed in the four evolutionary categories of DE genes. This illustrates, as in other phytophagous insects, the variety of biological processes involved in host specialization and their complex evolution combining plasticity, adaptation and species divergence [12, 20, 54, 56].

### A variety of biological and molecular functions involved in host specialization

Our experiment first confirmed the major role of digestive and detoxifying enzymes in host specialization as recurrently observed in various phytophagous insects [12]. Proteases, lipases, glucose dehydrogenases and sugar transporters were identified among the DE genes in the four A-D categories, highlighting the importance of these metabolic activities in plasticity and divergence. Serine proteases, in particular, were highly represented (Additional file 3: Table S4). These enzymes are thought to play a key role in lepidopterans having to deal with proteinase inhibition by the host plant [18, 52, 53, 55] and in defenses against pathogenic fungi in *Ostrinia furnacalis,* the Asian corn borer [57]. Genes encoding other enzymes, such as cytochrome P450, glutathione S-transferases, UDP-glycosyl transferases and carboxylesterases (Additional file 3: Table S4), were found to be differentially expressed between ECB and ABB and between maize and mugwort in either ECB or ABB. These enzymes may be involved in defenses against toxic plant metabolites, such as DIMBOA and MBOA in maize [58].

In addition, many cuticle-related genes were identified as displaying differentially expression between host plants (Additional file 3: Table S4) and in particular in the D category, which illustrate genes of differentiation between ECB and ABB due to host specialization. The chitin synthesis and degradation are the first key processes in larval development and molting. But chitin is also an integral component of the peritrophic matrix acting as a semipermeable barrier between the food bolus and the midgut, and protecting the larvae against toxins and pathogens [59]. Consequently some cuticle-related genes may play a key role in the insect-host relationship, in term of protection against both plant metabolites and pathogens. Transcripts for these proteins have repeatedly been identified in transcriptomic studies of the response of insects to various hosts [18, 54, 60–62]. In addition, immune genes (e.g. attacin, cecropin, gloverin, Additional file 3: Table S4) were found to be active and differentially expressed, particularly in mugwort samples. Finally, cuticular, detoxification and immune genes may all contribute to defend the caterpillar against pathogens and/or toxic plant compounds.

Finally, several odorant and gustatory receptors were differentially expressed in ECB and ABB, and some of these receptors were also differentially expressed between samples reared on maize and mugwort (B and D categories). In *Ostrinia* species, active chemosensory behavior is thought to occur mostly during the dispersal or mating phases (i.e. adult stage), and to be limited in larvae, which develop mostly on the plant on which they hatched, which was selected for egg mass laying by their mothers. The differences in chemosensory gene activity and expression observed here is in contradiction with the prevailing view, but are consistent with a previous study demonstrating sensing activity in ECB larvae [63]. In addition, the differential expression in larvae of six olfactory receptors (four of which were poorly expressed at the adult stage [64]) support their role in plant recognition.

### A potential role of associated microorganisms – Toward an enlarged ecological niche

In the present experiment, we observed a variety of DE genes homologous to microorganisms such as virus, yeast, bacteria and fungi. High levels of yeast and bacterial diversity have previously been reported in the ECB midgut and excrement [65, 66], and a clear relationship has been established between diet (artificial and host plant) and the composition of the midgut microbiota. Fine characterization of the complete microorganism community would be specious here, because our sequencing design did not target them specifically. Yet some of the genes of these microorganisms passed the statistical filter and tests (DE genes) and qualitative trends emerged that potentially link some of these microorganisms, and the metabolism and immune response of ECB and ABB. Overall, virus and bacteria DE genes were more observed in ECB samples, while yeast and fungi genes were more associated to ABB samples on maize. Interestingly, our results also suggested that the impact of some of these microorganisms on the metabolism and defense of ECB and ABB might be modulated by the plant diet, as illustrated by the differential activity of immune, detoxifying and cuticular genes and the lower survival of ABB larvae on mugwort. There is indeed increasing evidence for a strong effect of host plants (diet) on herbivore survival through the modulation of susceptibility to natural enemies [67]. For example, Knight & Witzgall [68] demonstrated that the combination of a pathogenic virus and mutualistic yeasts increased the mortality of *Cydia pomonella* larvae over that observed in the presence of the virus alone, suggesting that yeasts, viruses and host plants may affect larval survival through complex interactions. These studies, and our observations, advocate for the exploration of these multitrophic relationships to measure their role in the divergence by host specialization in phytophagous moths.

## Conclusions

We report here the first comprehensive survey of the transcriptomic responses of ECB and ABB larvae to different host plants. As in other comparable studies on other phytophagous insects, we observed an extensive transcriptomic response, involving processes of plant recognition, digestion and detoxification and genes involved in immunity or cuticular processes, worthy of further investigation. Our experimental and analytical design made it possible to classify genes according to their evolution and plasticity within and between ECB and ABB, yielding divergent and common, ancestral and derived patterns that it may subsequently be possible to relate to neutral and adaptive divergence between ECB and ABB. We also highlight the importance of associated microorganisms, which may play a crucial role in the insect-host relationship. This enlarged ecological niche opens up a promising avenue of research on the diversity of microorganisms *in natura*, their beneficial or pathogenic impact, and the mechanisms of defense or symbiosis involved in the ECB and ABB moths themselves.

Currently, the control of the maize pest ECB is mainly done with insecticide treatments and releases of biological control agents, both of which may impact non-target species. Our findings hence open up interesting new opportunities for pest management through targeted biopesticides [69] or gene manipulation via RNA interference [70]. The role of some candidate genes should now be investigated in other conditions (natural populations or other hosts) or at other stages of the lifecycle. For example, the chemosensory genes shown here to be expressed at the larval stage, may play a role in oviposition choice in adults, for which preferences differ strongly between female adults of ECB and ABB [26].

## Additional files

Additional file 1: **Table S1.** Male / female proportions in sequenced RNA pools; **Table S2.** Read mapping percentages on ECB-ref and ABB-ref; **Table S5.** Enriched GO terms in evolutionary A-D categories (Figs. 1 & 5), after Fisher’s exact test in Blast2GO and for a FDR < 0.05; **Figure S1.** Number of expressed transcripts per experimental set-up (moth species x host plant). A. Venn diagram for ECB-ref transcripts B. Venn Diagram for ABB-ref transcripts; **Figure S2.** Heatmaps of DE genes in ECB-ref by evolutionary category: (S2-A) differential expression (DE) between plants and not between moth species, (S2-B) DE between moth species and not between plants, (S2-C) DE between moth species and between plants, parallel trend, (S2-D) DE between moth species and between plants, opposite trend. (PDF 285 kb)

Additional file 2: **Table S3.** Common and private DE genes in analyses with and without the inclusion of the one outlier sample (ECB_Mug2). (XLSX 403 kb)

Additional file 3: **Table S4.** Detailed information for all DE genes: mean normalized counts per experimental set-up, significant effects in the GLM model (host plant, moth species, interaction) and functional annotations. (XLSX 1085 kb)

Additional file 4: **Table S6.** Detailed information for DE genes displaying significant homology to genes described in viruses, yeasts and fungi: mean normalized counts per experimental set-up, model effects (host plant, moth species, interaction) and functional annotations. (XLSX 45 kb)

Additional file 5: **Table S7.** Detailed information for DE genes displaying significant sequence similarity to genes previously described in bacteria: mean normalized counts per experimental set-up, model effects (host plant, moth species, interaction) and functional annotations. (XLSX 46 kb)

## Abbreviations

ABB: Adzuki bean borer
bp: Base pairs
DE: Differentially expressed
ECB: European corn borer
FDR: False discovery rate
GLM: Generalized linear model
GO: Gene ontology
L4: Fourth larval instar
LRT: Likelihood ratio test
Mb: Mega base pairs
MDS: Multidimensional scaling
TMM: Trimmed mean M values
