# Supplementary material for: Larval transcriptomic response to host plants in two related phytophagous lepidopteran species: implications for host specialization and species divergence
Source: BMC Genomics. 2018 Apr 18;19:265. doi: 10.1186/s12864-018-4589-x (PMC5907310; doi:10.1186/s12864-018-4589-x)
Supplement: Supplementary file 1 — Table S1. Male / female proportions in sequenced RNA pools; Table S2. Read mapping percentages on ECB-ref and ABB-ref; Table S5. Enriched GO terms in evolutionary A-D categories (Figs. 1 & 5), after Fisher’s exact test in Blast2GO and for a FDR < 0.05; Figure S1. Number of expressed transcripts per experimental set-up (moth species x host plant). A. Venn diagram for ECB-ref transcripts B. Venn Diagram for ABB-ref transcripts; Figure S2. Heatmaps of DE genes in ECB-ref by evolutionary category: (S2-A) differential expression (DE) between plants and not between moth species, (S2-B) DE between moth species and not between plants, (S2-C) DE between moth species and between plants, parallel trend, (S2-D) DE between moth species and between plants, opposite trend. (PDF 285 kb) [file 12864_2018_4589_MOESM1_ESM.pdf]

Supporting Information

Larval transcriptomic response to host plants in two related phytophagous lepidopteran species: implications for host specialization and species divergence.

Orsucci M.[1,2,5], Audiot P.[1], Dorkeld F.[1], Pommier A.[1], Vabre M.[3], Gschloessl B.[1], Rialle S[4], Severac D[4], Bourguet D.[1], Streiff R [1,2]

Table S1. Male / female proportions in sequenced RNA pools.

Pool ID	Set-up	Rep.	*n*	*n* females	*n* males	*n* undetermined
**1**	ECB on mugwort	1	20	11	9	
**2**	ECB on mugwort	2	20	10	10	
**3**	ECB on mugwort	3	20	10	10	
**4**	ECB on maize	1	19	11	7	1
**5**	ECB on maize	2	20	10	10	
**6**	ECB on maize	3	20	12	8	
**7**	ABB on mugwort	1	20	9	11	
**8**	ABB on mugwort	2	20	9	11	
**9**	ABB on mugwort	3	20	6	13	1
**10**	ABB on maize	1	20	8	12	
**11**	ABB on maize	2	20	13	6	1
**12**	ABB on maize	3	20	5	15	

'Rep' is the replicate ID for each experimental set-up. '*n*' indicates the total number of L4 larvae per pool. 'undetermined' corresponds to samples with ambiguous patterns after molecular sexing. We observed a 1:1 sex ratio for all but one pool (pool ID:12), which was significantly biased towards males (binom.test, $P = 0.04$).

Table S2. Read mapping percentages on ECB-ref and ABB-ref

ID pool samples	Experimental set-up	*n* reads	% of mapped reads (% of multiple mappings)	
			**ECB-ref**	**ABB-ref**
1	ECB on mugwort	40 013 932	68.41 (0.87)	66.15 (0.34)
2	ECB on mugwort	42 897 548	67.75 (0.98)	64.96 (0.31)
3	ECB on mugwort	37 588 781	68.02 (0.95)	65.60 (0.36)
4	ECB on maize	35 075 026	68.61 (0.91)	66.38 (0.34)
5	ECB on maize	31 352 629	67.77 (0.96)	64.64 (0.32)
6	ECB on maize	33 469 854	67.59 (0.93)	64.57 (0.31)
7	ABB on mugwort	50 384 518	67.45 (1.04)	65.00 (0.36)
8	ABB on mugwort	46 415 261	68.16 (0.87)	66.65 (0.31)
9	ABB on mugwort	40 448 627	69.06 (0.92)	67.03 (0.29)
10	ABB on maize	40 365 441	68.10 (0.94)	66.18 (0.35)
11	ABB on maize	42 563 625	69.03 (0.94)	67.14 (0.37)
12	ABB on maize	35 307 771	64.57 (0.84)	67.17 (0.36)

Table S5. Enriched GO terms in evolutionary A-D categories (Figures 1 & 5), after Fisher's exact test in Blast2GO and for a FDR <0.05.

Category	GO category	Enriched GO terms	O/U
A. Plant effect	∅	∅	∅
B. Moth species effect	BIOLOGICAL PROCESS	GO:0006259 DNA metabolic process	O
		GO:0071897 DNA biosynthetic process	O
		GO:0006278 RNA-dependent DNA biosynthetic process	O
		GO:0090304 nucleic acid metabolic process	O
		GO:0015074 DNA integration	O
		GO:0006139 nucleobase-containing compound metabolic process	O
		GO:1901360 organic cyclic compound metabolic process	O
		GO:0006725 cellular aromatic compound metabolic process	O
		GO:0046483 heterocycle metabolic process	O
		GO:0034641 cellular nitrogen compound metabolic process	O
		GO:0034654 nucleobase-containing compound biosynthetic process	O
		GO:0006807 nitrogen compound metabolic process	O
		GO:0019438 aromatic compound biosynthetic process	O
		GO:0018130 heterocycle biosynthetic process	O
		GO:1901362 organic cyclic compound biosynthetic process	O
		GO:0043170 macromolecule metabolic process	O
		GO:0044271 cellular nitrogen compound biosynthetic process	O
		GO:0044699 single-organism process	U
		GO:0044260 cellular macromolecule metabolic process	O
		GO:0044238 primary metabolic process	O
		GO:0009059 macromolecule biosynthetic process	O
		GO:0050789 regulation of biological process	U
		GO:0044267 cellular protein metabolic process	U
		GO:0034645 cellular macromolecule biosynthetic process	O
		GO:0050794 regulation of cellular process	U
		GO:0071704 organic substance metabolic process	O
		GO:0044763 single-organism cellular process	U
		GO:0044249 cellular biosynthetic process	O
		GO:1901576 organic substance biosynthetic process	O
		GO:0044237 cellular metabolic process	O
	MOLECULAR FUNCTION	GO:0034061 DNA polymerase activity	O
		GO:0016779 nucleotidyltransferase activity	O
		GO:0003964 RNA-directed DNA polymerase activity	O
		GO:0016772 transferase activity, transferring phosphorus-containing groups	O
		GO:0097367 carbohydrate derivative binding	U
		GO:0008270 zinc ion binding	O
		GO:0001882 nucleoside binding	U
		GO:0001883 purine nucleoside binding	U
		GO:0017076 purine nucleotide binding	U
		GO:0032553 ribonucleotide binding	U
		GO:0032555 purine ribonucleotide binding	U
		GO:0032550 purine ribonucleoside binding	U
		GO:0032549 ribonucleoside binding	U
		GO:0003676 nucleic acid binding	O
		GO:0035639 purine ribonucleoside triphosphate binding	U
		GO:0032559 adenyl ribonucleotide binding	U
		GO:0030554 adenyl nucleotide binding	U
		GO:0005524 ATP binding	U
		GO:0046914 transition metal ion binding	O
		GO:0043168 anion binding	U
	CELLULAR COMPONENT	GO:0005623 cell	U
		GO:0044464 cell part	U
		GO:0005622 intracellular	U
		GO:0016020 membrane	U
		GO:0044424 intracellular part	U
		GO:0043229 intracellular organelle	U
		GO:0043226 organelle	U
C. Moth species and plant effect (no interaction)	MOLECULAR FUNCTION	GO:0045735 nutrient	O
D. Moth species and plant effect (interaction)	∅	∅	∅

A.

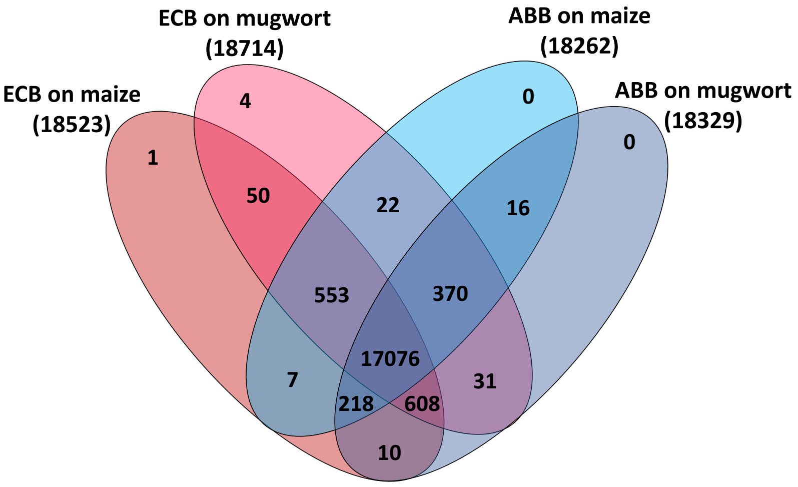

B.

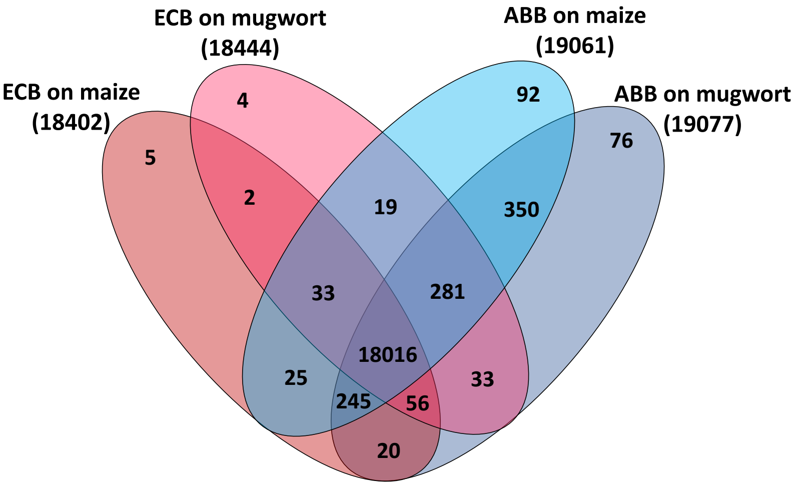

Figure S1: Number of expressed transcripts per experimental set-up (moth species x host plant). **A.** Venn diagram for ECB-ref transcripts **B**. Venn Diagram for ABB-ref transcripts.

S2-A.Plant effect

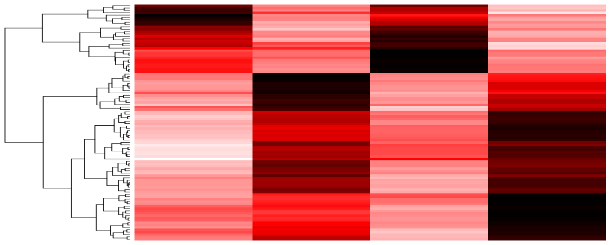

S2-B. Moth species effect

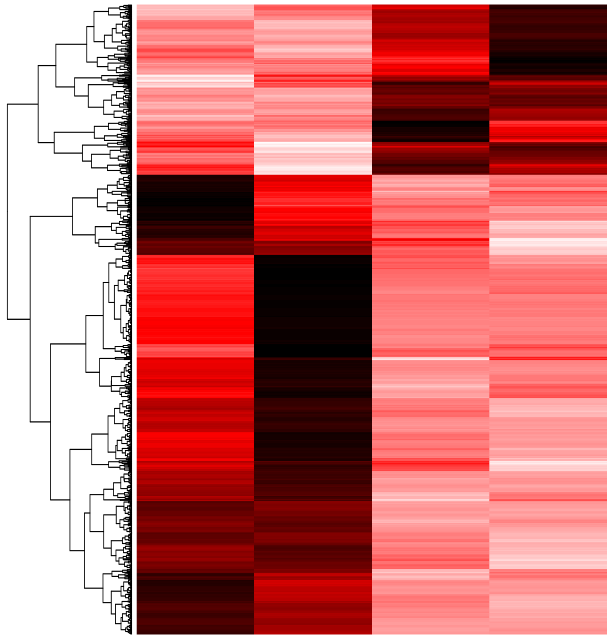

S2-C. Moth species and plant effect, no interaction

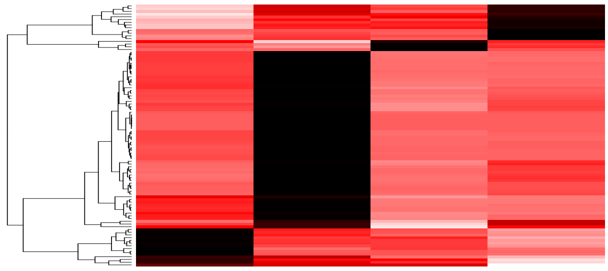

S2-D. interaction, opposite direction

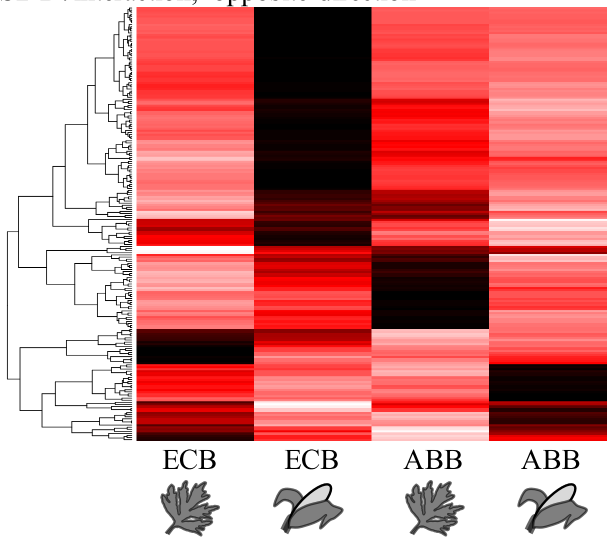

Figure S2: Heatmaps of DE genes in ECB-ref by evolutionary category: (**S2-A**) differential expression (DE) between plants and not between moth species, (**S2-B**) DE between moth species and not between plants, (**S2-C**) DE between moth species and between plants, parallel trend, (**S2-D**) DE between moth species and between plants, opposite trend**.** Genes (rows) were clustered with the 'hclust' algorithm of the 'heatmap' function in R. A white-red-black color scale indicates the low-middle-high expression of the genes.
